# Multiple Behavior Phenotypes of the Fragile-X Syndrome Mouse Model Respond to Chronic Inhibition of Phosphodiesterase-4D (PDE4D)

**DOI:** 10.1038/s41598-017-15028-x

**Published:** 2017-11-07

**Authors:** Mark E. Gurney, Patricia Cogram, Robert M Deacon, Christopher Rex, Michael Tranfaglia

**Affiliations:** 1 Tetra Discovery Partners, Inc, Grand Rapids, MI USA; 2 FRAXA-DVI, FRAXA, Santiago, Chile; 3 Laboratory of Molecular Neuropsychiatry, Institute of Cognitive and Translational Neuroscience (INCyT), INECO Foundation, Favaloro University, National Scientific and Technical Research Council, Buenos Aires, Argentina; 4 IEB, Faculty of Science, University of Chile, Santiago, Chile; 5 Afraxis, Inc, San Diego, CA USA; 6 FRAXA Research Foundation, Newburyport, MA USA

## Abstract

Fragile-X syndrome (FXS) patients display intellectual disability and autism spectrum disorder due to silencing of the X-linked, fragile-X mental retardation-1 (*FMR1*) gene. Dysregulation of cAMP metabolism is a consistent finding in patients and in the mouse and fly FXS models. We therefore explored if BPN14770, a prototypic phosphodiesterase-4D negative allosteric modulator (PDE4D-NAM) in early human clinical trials, might provide therapeutic benefit in the mouse FXS model. Daily treatment of adult male *fmr1* C57Bl6 knock-out mice with BPN14770 for 14 days reduced hyperarousal, improved social interaction, and improved natural behaviors such as nesting and marble burying as well as dendritic spine morphology. There was no decrement in behavioral scores in control C57Bl6 treated with BPN14770. The behavioral benefit of BPN14770 persisted two weeks after washout of the drug. Thus, BPN14770 may be useful for the treatment of fragile-X syndrome and other disorders with decreased cAMP signaling.

## Introduction

Mutational inactivation of the gene encoding the Fragile X Mental Retardation protein (FMRP) causes a spectrum of symptoms including seizures, sleep disorders, anxiety, irritability, autism, mild to severe cognitive impairment and intellectual disability^1,2^. The constellation of symptoms is known as Fragile-X syndrome (FXS). The syndrome in humans is caused by expansion of an unstable, CGG triplet repeat with subsequent silencing of the fragile-X mental retardation-1 (*FMR1*) gene that encodes FMRP. FXS is a common monogenic disorder that affects 1 in 3,600 males and 1 in 4,000–6,000 females^2^. FXS is severely debilitating in males^3^. Females generally are less affected than males due to mosaicism resulting from X-chromosome inactivation which occurs randomly early in embryogenesis.

Present both pre- and post-synaptically, FMRP associates with and suppresses the translation of mRNA important for synaptic function^4,5^. Consequently, genetic ablation of FMRP allows the over expression of proteins that normally would be tightly regulated components of the synapse such as channels, signaling and structural components. The syndrome can be modeled in mice and fruit flies by deletion of the *fmr1* gene in mice and the highly conserved *Dfmr1* gene in *Drosophila* ^6,7^.

Initial findings supporting phosphodiesterase-4 (PDE4) as a therapeutic target in FXS come from work in *Drosophila* ^8,9^. Interest in PDE4 derived from the observations by Berry-Kravis and coworkers of reduced cAMP levels in FXS patient cells^10,11^. *Drosophila* has a single, *FMR1* gene otholog that when deleted produces deficits in neuronal development and biochemical and behavioral changes reminiscent of human FXS^7,12,13^. *Dfmr1* null flies have impaired associative memory in an olfactory conditioning paradigm and structural alterations in mushroom body neurons, a neural center important for associative learning, accompanied by decreased cAMP in tissues of the head^8,9,14,15^. Using a *Drosophila* model in which the flies are heterozygous for the *Dmfr1* ^3^ null allele, Kanellopoulos and coworkers reported that treatment with rolipram, a PDE4 inhibitor, rescued the olfactory learning deficit^9^. The learning deficit also was rescued by crossing the *Dmfr1* ^3^ allele onto a genetic background lacking the fly *dunce* gene (*Dnc*), the single *Drosophila* PDE4 gene. These findings were extended by Choi and coworkers to a *Drosophila* model in which *Dmfr1* was completely absent^8^. Two PDE4 inhibitors, rolipram and RO201724, were shown to reverse the behavioral deficits in *Dmfr1* null flies. A low dose of rolipram did not rescue the structural abnormalities in the mushroom body neurons, while a high dose rescued both behavioral and structural phenotypes. Choi and coworkers also showed genetic rescue of the *Dmfr1* null behavioral and structural phenotypes on the *dunce* background. Thus, reducing PDE4 activity in the *Drosophila Dmfr1* models rescues multiple aspects of the Fragile-X phenotype.

While the *Drosophila* genome contains a single PDE4 gene, this has been expanded to a small gene family in higher organisms. The genomes of humans and other mammals contain four PDE4 genes (PDE4A-D)^16^. The gene family contains two upstream conserved regions (UCR1 & UCR2) important for regulation of PDE4 enzymatic activity that distinguish the PDE4 enzymes from other PDE. UCR1 and UCR2 are ancestral domains that are conserved in *Drosophila* and *Caenorhabditis elegans* but not in *Dictyostelium* or yeast^17^. Each gene expresses multiple proteins that differ in N-terminal targeting sequences, their assembly into dimeric or monomeric forms of the PDE4 enzyme, and their post-translation regulation through protein kinase A (PKA) phosphorylation^18,19^. The importance of PDE4D for human cognition is shown by ultra-rare, autosomal dominant mutations in PDE4D that cause acrodysostosis without hormone resistance (ACRDYS2), a neurodevelopmental syndrome causing short stature, brachydactyly (short fingers and toes), nasal hypoplasia and intellectual disability with speech and psychomotor retardation^20,21^. All of the ACRDYS2 mutations described to date are missense mutations that alter amino acids on the surface of the protein such as the contact residues between the PDE4D catalytic domain and the UCR2 regulatory domain^20,22–27^. One mutation (serine129 to alanine) removes the PKA phosphorylation site on the UCR1 regulatory domain, and therefore prevents activation of PDE4D enzymatic activity in response to cAMP signaling. The implication is that dysregulation of the spatial and temporal patterning of cAMP signaling by reducing cAMP hydrolysis, as in *Drosophila dunce* mutant flies, impairs associative memory^28^. PDE4D negative allosteric modulators (PDE4D-NAM) such as BPN14770 inhibit the enzyme by closing the UCR2 regulatory domain across the active site, thereby limiting access of cAMP^29^. Unlike rolipram and RO201724, which inhibit all subtypes of PDE4, BPN14770 is selective for the PDE4D subtype. We therefore sought to assess the therapeutic benefit of BPN14770 in adult, male *fmr1* gene deleted mice in order to extend previous studies in the *Drosophila* FXS model.

FXS patients display a range of neuropsychiatric symptoms including intellectual disability, delayed language acquisition, poor social interaction, hyperarousal, hypersensitivity, repetitive behaviors, disrupted sleep, attention deficit hyperactivity disorder and autism^2^. These behavioral changes are modeled in adult male *fmr1* KO mice which display a spectrum of behavioral phenotypes due to the *fmr1* gene deletion^6^. The mutant mice show hyperarousal in the open field test, have impaired social interaction, are less likely to build nests when provided cotton batting and are less likely to bury marbles in the cage bedding. Adult male mice were used for all studies as male FXS patients typically suffer more severe symptoms than do female patients due to the single X chromosome^2^. In both FXS patients and the *fmr1* KO mice, there are alterations in the density, size, shape and maturity of dendritic spines, the principle recipients of excitatory inputs from other neurons^30,31^.

When treated daily with the PDE4D inhibitor BPN14770 for 14 days, *fmr1* KO mice showed significantly reduced hyperarousal in the open field, significantly increased frequency of social interaction, and significantly improved natural behaviors (nesting and marble burying) in comparison to *fmr1* KO mice treated with vehicle. Indeed, measures of activity, social interaction, nesting and marble burying were not significantly different from wild-type C57Bl6 mice. There was no decrement in behavioral scores in control C57Bl6 treated with BPN14770. The benefit of BPN14770 persisted two weeks after washout of the drug. The present study supports PDE4D as a therapeutic target for the treatment of FXS. BPN14770 has completed human Phase 1 clinical testing with evidence of safety and tolerability. Our results suggest that BPN14770 may be useful for the treatment of fragile-X syndrome with potential expansion to the treatment of autism spectrum disorder.

## Results

### BPN14770 Improves Behavioral Phenotypes of *fmr1* KO Mice

**Figure 1:**
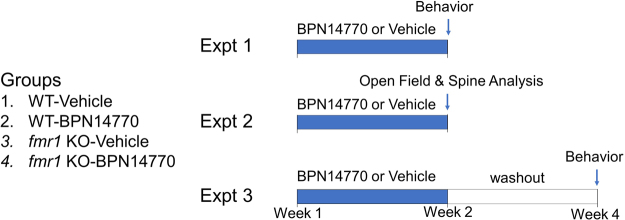
Schematic Diagram Depicting Treatment Groups and Schedule of Events. Treatment groups were wild-type C57Bl6 mice exposed to vehicle (WT-Vehicle), wild-type C57Bl6 mice exposed to BPN14770 (WT-BPN14770), *fmr1* KO mice exposed to vehicle, or *fmr1* KO mice exposed to BPN14770. Three experiments were performed. In each experiment, mice were treated daily for 14 days by oral gavage with either vehicle or BPN14770. In the first experiment (Expt 1), mice were profiled for open field activity, social interaction, and natural behaviors (nesting & marble burying) on the 14^th^ day of treatment. In the second experiment (Expt 2), mice were treated daily for 14 days, profiled for open field activity on the 14^th^ day of treatment, then killed and prepared for spine morphometry. In the third experiment (Expt 3), mice were treated daily for 14 days, treatment was then stopped, and 14 days after cessation of treatment the mice were profiled for open field activity, social interaction, nesting & marble burying. Each treatment group contained 10 adult male mice.

The design of the study is shown in Fig. 1. Four groups of mice were dosed in each experiment. The groups were wild-type C57Bl6 mice dosed orally with vehicle (WT-Veh), wild-type mice dosed with BPN14770 (WT-BPN14770), *fmr1* gene deleted or knock-out (KO) mice dosed with vehicle (*fmr1* KO-Veh), and *fmr1* KO mice dosed with BPN14770 (*fmr1* KO-BPN14770). Mice were dosed orally with vehicle or BPN14770 once daily for 14 days. BPN14770 was dosed at 0.3 mg/kg PO in 0.5% methylcellulose. This was 3 times the minimum effective oral dose (0.1 mg/kg) found in separate studies to improve novel object recognition after a 24 hr delay in ICR mice (unpublished data). Three separate experiments with *fmr1* KO mice were performed sequentially. In the first experiment, the groups of mice were dosed for 14 days and behavioral assessments were performed 2 hours after the last dose on day 14. In the second experiment, mice were dosed for 14 days, the open field test was performed 2 hour after the last dose on day 14, and then the mice were killed and the brains were prepared for spine morphometry. In the third experiment, mice were dosed for 14 days, the drug was withdrawn, and then behavioral assessments were performed 14 days after the last dose of drug or vehicle.

**Figure 2:**
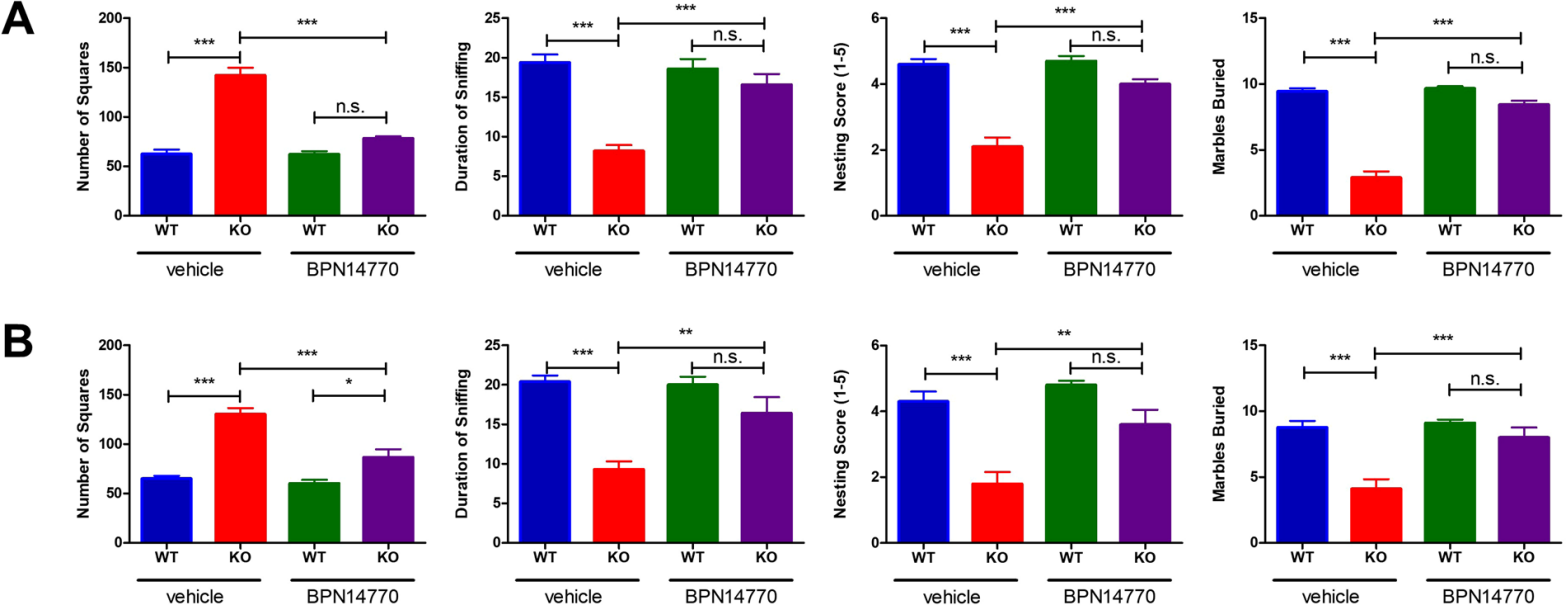
BPN14770 Improved Behavioral Phenotypes of *fmr1* KO Mice **(A)** Wild-type (WT) or *fmr1* KO (KO) adult male mice were treated with vehicle or BPN14770 daily for 14 days (Expt 1) and then profiled for behavior. From left-to-right, data are shown for hyperarousal (Number of Squares crossed in the open field test), social interaction (Duration of Sniffing), nesting (Nesting Score), and marble burying (Marbles Buried). **(B)** Mice were treated daily for 14 days with vehicle or BPN14770 and then drug was withdrawn for 14 days (Expt 3). Mice were profiled for hyperarousal, social interaction, nesting and marble burying on the 14^th^ day after drug withdrawal. Data were analyzed by Two-way ANOVA followed by Tukey’s post-test corrected for multiple comparisons. P-values shown are: n.s. not significant, *p < *0.05*, **p < *0.01*, ***p < *0.001* (*N* = 10 mice per group).

BPN14770 improved a spectrum of behavioral deficits in adult male *fmr1* KO mice (Fig. 2). For behavioral assessment, mice were randomized based on genotype to groups treated with vehicle or BPN14770. Based on two-way ANOVA, there was a highly significant interaction between treatment and genotype (*F*_(1,36)_ = 41.78, *p* < *0.001*). *Fmr1* C57Bl6 knock-out mice treated with vehicle were significantly more active in the open field than wild type C57Bl6 mice treated with vehicle (*p* < *0.001*). *Fmr1* C57Bl6 knock-out mice treated with BPN14770 for 14 days at 0.3 mg/kg showed significantly reduced open field activity in comparison to vehicle treated *fmr1* knock-out mice (*p* < *0.001*). There was no effect of BPN14770 on open field activity of wild-type mice, and indeed, after treatment with BPN14770, the open field activity of *fmr1* knock-out mice was not significantly different from wild-type mice treated with either BPN14770 or vehicle. Safety studies have shown no effect of BPN14770 on locomotion in C57Bl6 mice up to 30 mg/kg. Toxicological studies in rats identify the No Observed Effect Level (NOEL) of BPN14770 as greater than 60 mg/kg based on inappetence and weight loss. Thus, the reduction in open field activity of *fmr1* knock-out mice caused by BPN14770 at the low dose explored is unlikely to be due to an adverse effect of the drug.

Social interaction was assessed as the duration of sniffing when the *fmr1* KO mouse was tested with a juvenile mouse. There was a highly significant interaction between treatment and genotype (*F*_(1,36)_ = 17.05, *p* < *0.001*). The *fmr1* KO mice treated with vehicle showed a significantly reduced duration of sniffing of the juvenile mouse (*p* < *0.001*) compared to wild type C57Bl6 mice treated with vehicle. These social interaction deficits are consistent with those reported by other researchers^32^. *Fmr1* KO mice treated with BPN14770 showed increased duration of sniffing (*p* < *0.001*) that was not significantly different from wild-type mice. There was no effect of BPN14770 on the behavior of wild-type C57Bl6 mice.

Both male and female mice will build nests for thermoregulation when given access to nesting material in their home cage. This test has been used as an indicator of hippocampal lesion and dysfunction^33^. To assess nesting, mice were placed individually into the nesting cages about one hour before the dark phase, and the results were assessed the next morning. As with burrowing, and for similar reasons, timing was not critical. Nest building was scored on a 5-point scale and the amount of unturned nestlets was also weighed. As a reference, most C57BL/6 mice score 4–5 on nest construction, but when the hippocampus is lesioned the median score would be around 1–2 and a score of 3 is unlikely to be exceeded^33^. There was a highly significant interaction between treatment and genotype (*F*_(1,36)_ = 21.76, *p* < *0.001*). Nesting was significantly impaired in vehicle-treated *fmr1* C57Bl6 KO as compared to wild type C57Bl6 mice treated with vehicle (*p* < *0.001*). *Fmr1* C57Bl6 KO mice treated with BPN14770 showed significant improvement in nest building in comparison to vehicle treated *fmr1* KO mice (*p* < *0.001*) and indeed, were not significantly different from control C57Bl6 mice treated with vehicle. There was no effect of BPN14770 on nest building by wild-type C57Bl6 mice.

Mice spontaneously dig in many substrates in the laboratory. This behavior comes from their ancestry in the wild, where they forage for seeds, grain, insects, and other food to be found buried in the soil or leaf litter in their natural habitat. It exploits a common natural rodent behavior and provides quantitative data under controlled laboratory conditions. There was a highly significant interaction between treatment and genotype (*F*_(1,36)_ = 69.82, *p* < *0.001*). Marble burying was significantly impaired in vehicle- treated *fmr1* C57Bl6 KO as compared to wild-type C57Bl6 mice treated with vehicle (*p* < *0.001*). *Fmr1* C57Bl6 KO mice treated with BPN14770 showed significant improvement in marble burying in comparison to vehicle treated *fmr1* KO mice (*p* < *0.001*) and were not significantly different from control C57Bl6 mice. There was no effect of BPN14770 on marble burying by wild-type C57Bl6 mice.

Thus, treatment with BPN14770 reversed a range of behavioral phenotypes in the *fmr1* KO mutant mice.

### BPN14770 Improves Dendritic Spine Morphology in *fmr1* KO Mice

**Figure 3:**
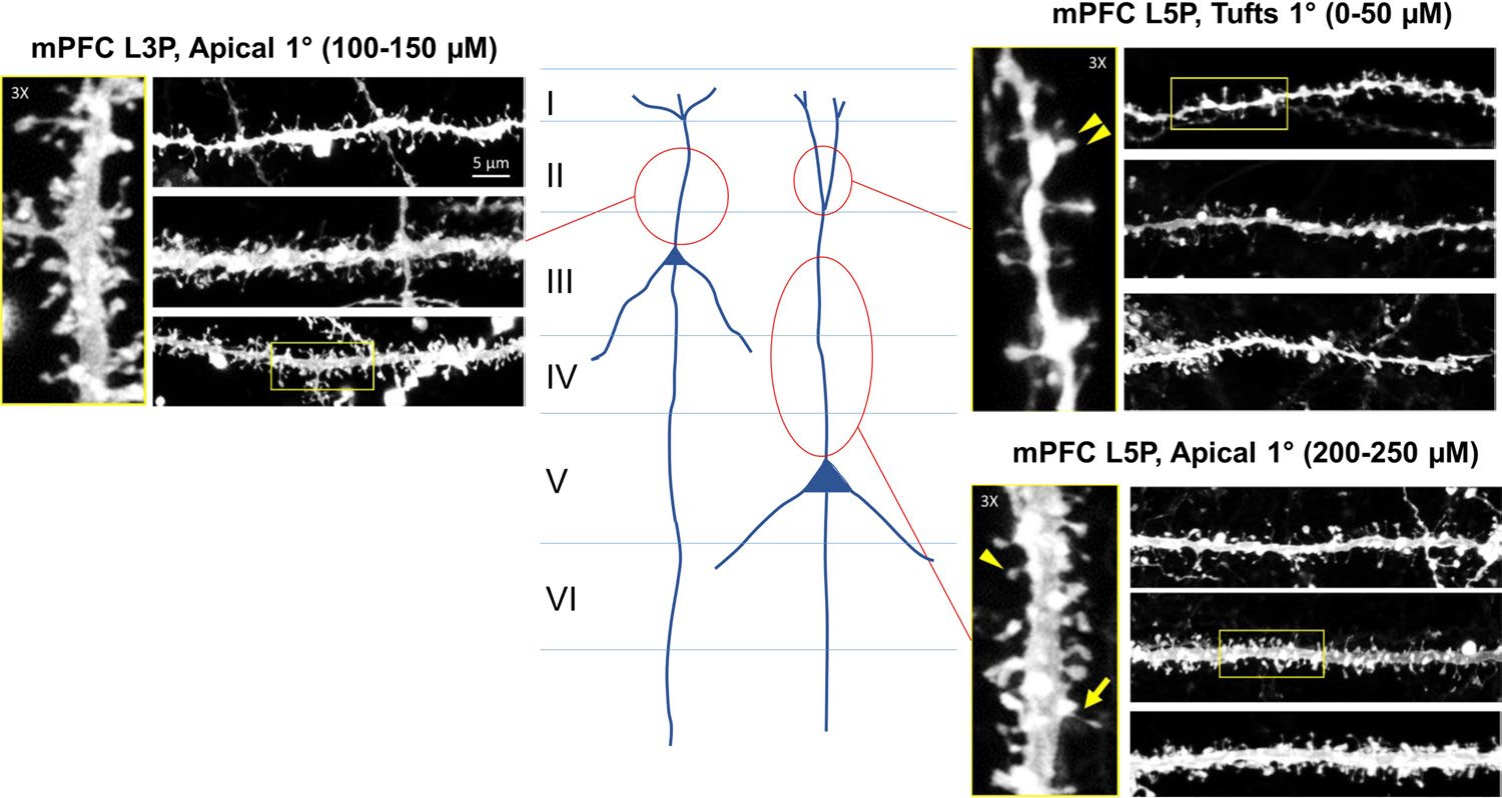
Representative Laser Scanning Confocal Micrographs Of Dendritic Segments And Dendritic Spines For Pyramidal Neurons In Medial Prefrontal Cortex. Dendritic spine morphometry was performed on dendritic segments of layer 3 and layer 5 pyramidal cells in medial prefrontal cortex (schematic). The sampling region for layer 3 pyramidal cells (L3P) was a segment of the primary apical dendrite extending from the soma to a distance of 100–150 µM. Layer 5 pyramidal cells were sampled from the soma to a distance of 200–250 µM of the primary apical dendrite and from the branch point of the apical tuft for a distance of 50 µM. High magnification insets for each segment sampled show the presence of thin-necked, immature spines (arrow) as well as mushroom (arrowhead only) and stubby spine (double arrowhead) types.

Dendritic spine morphology is altered in human FXS and in the fmr1 KO mouse model^34,35^. There is an abundance of spines with a thin, elongated morphology rather than a mature, squat, bulbous morphology. To assess if BPN14770 affected dendritic spine morphology, adult male *fmr1* KO mice were dosed daily with BPN14770 or vehicle for 14 days. When tested in the open field 2hrs after the last dose, mice treated with BPN14770 showed reduced hyperarousal (*p* < *0.001*) consistent with the behavioral findings in the first experiment (Supplementry Fig. 1). The mice were killed and prepared for spine morphometry. The spine morphometry analysis focused on medial prefrontal cortex, a region of cortex in which spine morphology is altered by the *fmr1* mutation (Fig. 3)^31,34,35^. Layer 3 and layer 5 pyramidal cells were chosen for morphometry. Immunohistochemical studies in mice indicate that PDE4D is mostly present in neurons of cortical layers 2 and 3^36^. Spines were sampled on the apical dendrite of layer 3 pyramidal cells and at two sites on layer 5 pyramidal neurons, on the apical dendrite and on the apical tufts.

**Figure 4:**
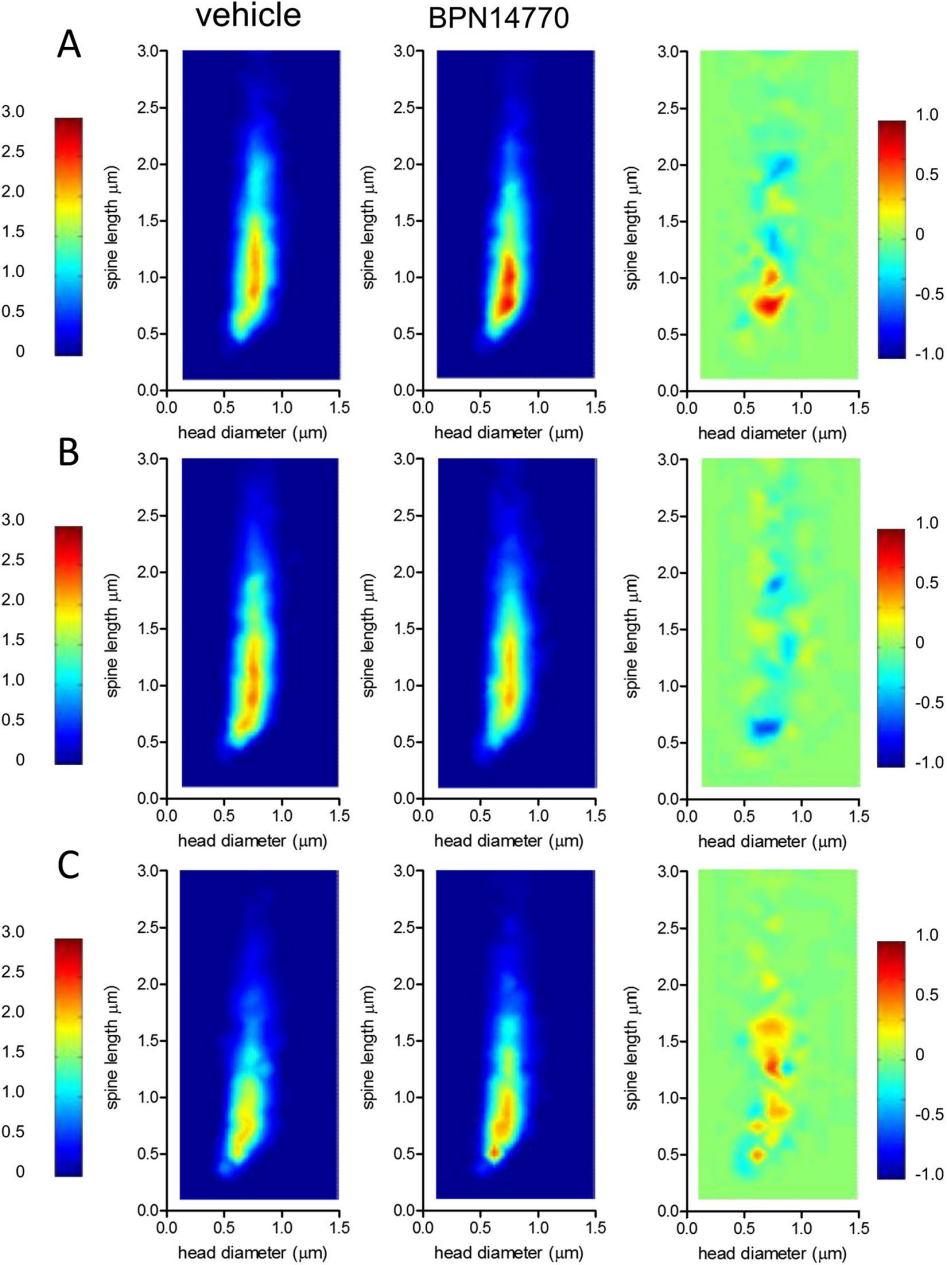
Dendritic Spine Multi-Dimensional Feature Distribution Plots. Multi-dimensional spine length x head diameter plots are shown for (**A**) the apical dendrite of Layer 3 pyramidal cells (*N* = 3,502 spines, 70 cells vehicle; *N* = 3,322 spines, 69 cells treated), (**B**) the apical dendrite of Layer 5 pyramidal cells (*N* = 3,792 spines, 70 cells vehicle; *N* = 3,443 spines, 69 cells treated), and (**C**) the apical dendrite tufts of Layer 5 pyramidal cells (*N* = 2,626 spines, 65 cells vehicle; *N* = 3,026 spines, 68 cells treated). The first two panels compare spines from *fmr1* KO mice exposed to vehicle or BPN14770. The third panel shows the difference plot. The difference map (vehicle subtracted from BPN14770) shows maturation (shortening) of spines on the apical dendrites of layer 3 pyramidal neurons. There was no change in dendritic spine maturation on layer 5 pyramidal neurons.

**Figure 5:**
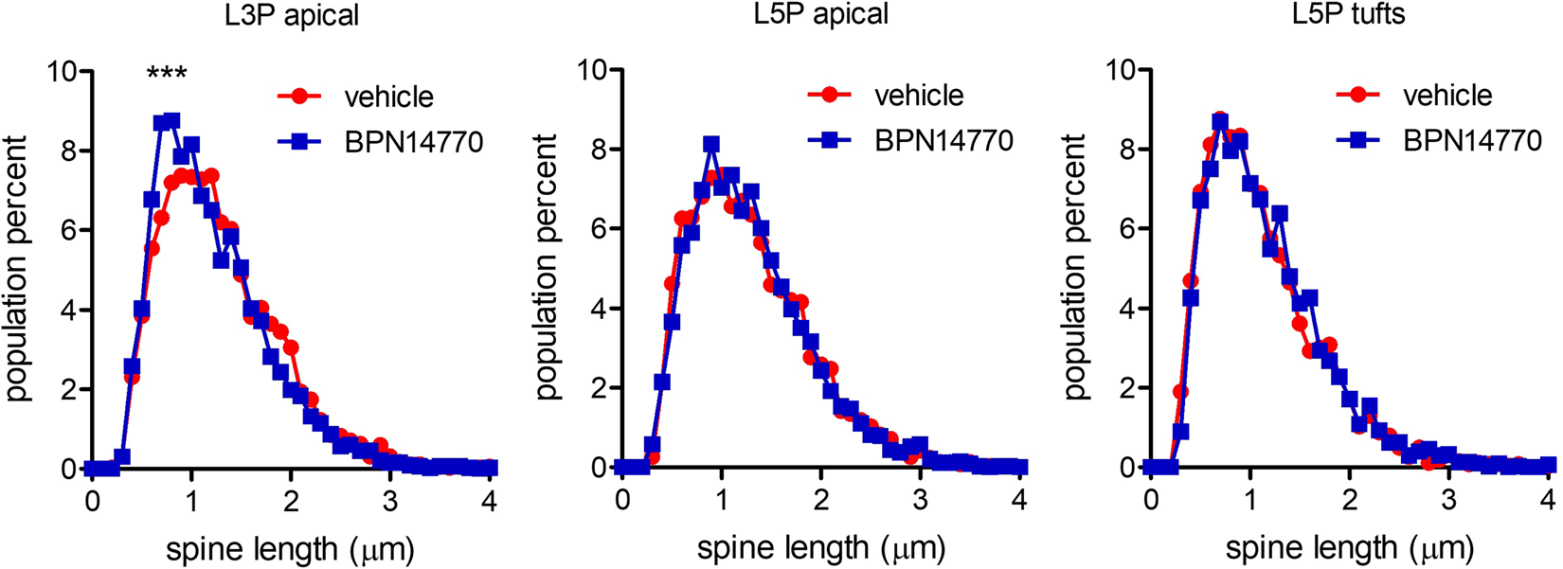
Effect of BPN14770 on Dendritic Spine Length. The distribution of spine length is shown for the apical dendrite of Layer 3 pyramidal cells (L3P apical), the apical dendrite of Layer 5 pyramidal cells (L5P apical), and the apical dendrite tufts of Layer 5 pyramidal cells (L5P tufts). Dendritic spines were sampled for length, head dimeter and neck width. Data are plotted for vehicle (red circles) and BPN14770-treated (blue squares) *fmr1* KO mice. Data were analyzed by the 2-sample Komolgorov-Smirnov test. P-values shown are ***p < 0.0001.

There was a highly significant effect of BPN14770 on the spine length of layer 3 pyramidal cells (p < 0.0001) which is consistent with an effect of BPN14770 on spine maturation (Figs 4 and 5). Treatment with BPN14770 causes reduction in layer 3 pyramidal cell spine length of about 10%. As an internal control, there was no effect of BPN14770 on spine length on layer 5 pyramidal cells on either the apical dendrite or the apical tufts.

The spine data also showed a trend by animal (*N* = 10 mice per group; *p* < *0.1*) for an increase in mature spines on layer 3 pyramidal cells after treatment with BPN14770, while there was no change in spine morphometry on layer 5 pyramidal neurons (Sup Fig. 2). When the data were analyzed by dendrite (*N* = 70 dendrites from mice treated with BPN14770, *N* = 69 dendrites from vehicle treated mice), there was a significant decrease in intermediate spines on layer 3 pyramidal cells (p < 0.05) and a trend for an increase in mature spines (*p* < *0.1*) (Sup Fig. 3). There was no effect of BPN14770 on the spines of layer 5 pyramidal neurons on either the apical dendrite or the apical tufts.

### Behavioral Benefit of BPN14770 Endures After Washout of the Drug

The third experiment explored whether or not the behavioral benefit of BPN14770 persisted after washout of the drug. Mice were treated daily with BPN14770 or vehicle for 14 days and then the drug was withdrawn for 14 days (Fig. 1). Even 14 days after withdrawal of the drug, *fmr1* KO mice previously treated with BPN14770 showed decreased hyperarousal in the open field (*p* < *0.001*), increased social interaction (*p* < *0.005*), improved nesting behavior (*p* < *0.005*) and increased marble burying (*p* < *0.001*). The activity of *fmr1* KO mice previously treated with BPN14770 was significantly greater than wild-type C57Bl6 mice treated with vehicle or BPN14770 (*p* < *0.05*) indicating that the improvement in behavior waned somewhat after washout of the drug.

## Discussion

Multiple drug classes have shown evidence of therapeutic efficacy in fragile X animal models; however, many have been validated via acute dosing in limited model systems. BPN14770 shows a broad range of therapeutic effects in *fmr1* KO mice with no loss of efficacy over an extended dosing period. The pro-cognitive effects of PDE4 inhibitors are potentially beneficial to humans with mosaic fragile X, a common presentation in males with the fragile X mutation and a universal condition in females with fragile X. The benefit of rolipram, an older PDE4 inhibitor, also strongly suggests class-wide antidepressant effects which could be useful in fragile X^37,38^. Other drug classes, such as GABA(B) agonists and mGluR5 NAMs are known to cause significant cognitive impairment and adverse psychiatric effects in human subjects^39,40^, suggesting that some agents with disease-specific effects in fragile X may be harmful to normal cells in a mosaic brain.

The cAMP dysregulation in FXS affects multiple tissues. In the original study by Berry-Kravis and Huttenlocher^41^, platelets from FXS patients were found to have lower basal and stimulated cAMP levels than controls. Basal, prostaglandin E1 and forskolin-stimulated cAMP levels were 50–60% of controls. In their experiment, a non-selective PDE inhibitor (1-isobutyl-3-methylxanthine) was used to inhibit all platelet PDE activity. This indicates that cAMP dysregulation is likely at the level of adenylate cyclase rather than due to dysregulation of a PDE. The dominant PDE in platelets is PDE3, the target of cilostazol, a selective PDE3 inhibitor indicated for the treatment of intermittent claudication in individuals with peripheral vascular disease^42^. PDE4 isoenzymes are absent from platelets^43^. Thus, it is unlikely that dysregulation of PDE4D could account for the alteration in cAMP levels seen in multiple tissues in FXS, nor is treatment with BPN14770 likely to normalize cAMP levels in all tissues. In brain, PDE4D is only one of three PDE4 subtypes expressed, the others being PDE4A and PDE4B, thus PDE4A, PDE4B and PDE4D all contribute to cAMP homeostasis^36,44,45^. At the low dose of BPN14770 used in this study (0.3 mg/kg), inhibition is still relatively selective for PDE4D, although the effect of BPN14770 on basal cAMP levels in *fmr1* KO mouse brain should be addressed in future studies.

Chronic dosing of 14 days duration with BPN14770 had broad efficacy across multiple behavioral measures in the *fmr1* mouse model of fragile X. In contrast to CTEP, an mGluR5 antagonist, BPN14770 improves social interaction and natural behaviors such as building nests and burying marbles^46^. Also in contrast to the mGluR5 antagonists, BPN14770 maintains efficacy with chronic dosing while the benefit of the mGluR5 antagonists may wane with repeat dosing^47^. Similarly, GABA(B) agonists have demonstrated acute efficacy in fragile X models, but chronic dosing leads to rapid development of tolerance^48^. In addition, unlike mGluR5 signaling, cAMP signaling has been shown to be altered in human fragile X tissues^10,11,49^ as well as multiple fragile X model systems^8,9^, providing an added level of validation for PDE4 as a therapeutic target in fragile X.

Chronic dosing with BPN14770 also improved spine morphology on layer 3 pyramidal cells in medial prefrontal cortex. As an internal control, there was no effect on the dendritic spines of layer 5 pyramidal cells. Layer 3 pyramidal cells are the principle input neurons for intracortical association while layer 5 pyramidal cells are the principle output cells. The selective effect of BPN14770 on layer 3 pyramidal neurons may be due to the preferential expression of PDE4D in this cortical cell type^36^. Such cells may be more responsive to PDE4D inhibition. Cruz-Martin *et al*. also has provided intriguing data showing that dendritic spine dynamics of layer 2/3 pyramidal neurons is altered in the barrel cortex of Fragile X knock-out mice^31^. Although pyramidal neurons were not probed in deeper layers of cortex, intracortical association neurons may be more plastic, and in adults, maturation of their dendritic spines may be more responsive to signaling through the PKA-CREB pathway and thereby to inhibition of PDE4D. Consistent with the effect of BPN14770 on cortical neuron structure, the behavioral benefit of BPN14770 endures well after washout of the drug. The half-life of BPN14770 in mice is 11 hr (unpublished data), therefore the enduring behavioral benefit is not due to slow washout of the drug.

These studies were conducted in adult male *fmr1* gene-deleted mice suggesting that adolescent and adult FXS patients may benefit from treatment with a PDE4D-NAM. One of the challenges in the design of clinical efficacy studies in FXS patients is the number of concomitant medications used to reduce symptoms of FXS, their impact on clinical aspects of the disorder that the novel neurotherapeutic may address, and the potential for pharmacokinetic as well as pharmacodynamic drug-drug interactions. FXS patients are prescribed multiple classes of psychotropic drugs including, but not limited to, antipsychotics to reduce irritability and aggression, serotonin reuptake inhibitors (SSRI) to reduce anxiety, and dopaminergic stimulants to reduce inattention and hyperactivity^3^. Younger patients are at higher risk for seizure and may be prescribed anticonvulsants. In contrast, when potential therapeutic agents are evaluated in animal models, either *Dfmr1* mutant flies or *fmr1* mutant mice, the agents are evaluated on a “clean” background in the absence of other psychotropic drugs. SSRIs are known to increase brain cAMP and PDE4D levels in preclinical models^50^, and indeed, upregulate PDE4 binding in patients with major depression^51^. Although SSRIs provide some control of anxiety in FXS patients, concomitant administration of an SSRI and a second drug that elevates cAMP may reduce the likelihood of discerning clinical benefit of the new agent on measures of anxiety or on clinical global impression scales incorporating anxiety in a composite measure.

In addition to FXS, there is a clear link between other monogenic disorders associated with intellectual disability and aberrant cAMP signaling through activation of PKA and consequent phosphorylation of the cAMP response element binding factor (CREB). CREB phosphorylation mediates the synaptic changes needed for the formation of various forms of memory^52–54^. Many of these disorders are amenable to study in model systems such as mice or *Drosophila*. Neurofibromatosis is linked to the cAMP pathway through the regulation by neurofibromin of adenylate cyclase production of cAMP with neuronal apoptosis phenotypes in an NF1 mouse model shown to be rescued by a PDE4 inhibitor^55^. Cognitive benefit was not examined in that study. CREB requires the cofactor CREB binding protein (CBP), a mutation in which causes Rubinstein-Taybi syndrome. Despite the mutation of CBP, PDE4 inhibitors are able to rescue memory phenotypes in a mouse model^56^. Finally, Rett syndrome, which is due to mutation of the MeCP2 gene, also affects CREB signaling by reducing CREB expression and phosphorylation by PKA, while activation of CREB signaling by a PDE4 inhibitor ameliorates multiple cellular and behavioral phenotypes^57^. Drugs in clinical development such as BPN14770 that target a specific molecular signaling pathway, as in the case of the cAMP-PKA-CREB pathway, may facilitate the new model of experimental medicine in which proof of concept studies in preclinical models and their translation to patients are used to interrogate specific biological processes and neurocognitive outcomes related to the drug mechanism of action.

## Methods

### Compounds

BPN14770 was synthesized as described^17^. The fifty percent inhibitory (IC_50_) potency of BPN14770 against mouse PDE4D is 133 ± 18 nM (mean + SEM) as compared to mouse PDE4B (IC_50_ = 2,124 ± 527 nM). BPN14770 does not inhibit other PDE (BPS Biosciences panel), GPCR, ion channels or transporters (CEREP panel) at 10 µM. BPN14770 has 100% bioavailability in mice when dosed by perioral gavage as a suspension in 0.5% methylcellulose.

### Animals

*Fmr1* KO2 C57Bl6 knock-out mice (The Dutch-Belgium Fragile X Consortium 1994)^6^ and wild-type (WT) littermates were generated on a C57BL/6J background and repeatedly backcrossed onto a C57BL/6J background for more than eight generations. Initial stocks of mice were obtained from the Jackson Laboratory. The *fmr1* KO2 is a null allele at *fmr1* generated by deletion of the promoter and first exon. It is both protein and mRNA null. This model is widely used by the FXS research community. The *fmr1* KO2 C57Bl6 KO mice were housed in groups of the same genotype in a temperature- and humidity-controlled room with a 12-hr light–dark cycle (lights on 7 a.m.–7 p.m.). Testing was conducted during the light phase. Food and water were available *ad libitum*. Testing was conducted on *fmr1* KO mice and their WT littermates. Ten male mice per treatment group, 14 weeks of age, were used for behavioral experiments. Mice were housed in commercial plastic cages, and experiments were conducted in accordance with the requirements of the UK Animals (Scientific Procedures) Act, 1986. Protocols were reviewed and approved by the Fraunhofer Chile Institute review board.

### Randomization and Blinding

All experiments were conducted with the Fraunhoeffer Institute staff blinded to genotype and drug treatment. Separate investigators prepared and coded the dosing solutions, allocated the mice to the study treatment groups, dosed the animals, and collected the behavioral data.

### Treatment Groups and Dosing

There were four treatment groups in the study with 10 male mice used per treatment group:

Group 1: *fmr*1 C57Bl6 knock-out mice treated with vehicle,

Group 2: wild-type C57Bl6 mice treated with vehicle,

Group 3: *fmr1* C57Bl6 knock-out mice treated with BPN14770,

Group 4: wild-type C57Bl6 mice treated with BPN14770.

Mice were dosed by perioral gavage using a gavage tube suitable for mice. BPN14770 was dosed at 0.3 mg/kg once daily for 14 days as a suspension in 0.5% methylcellulose in sterile water. The suspension was stirred or shaken to prevent settling and maintain homogeneity. The dosing solution was prepared fresh daily. Vehicle-treated mice were dosed orally with 0.5% methylcellulose in sterile water. Behavioral assessment was after 14 days of dosing (Expt 1 and Expt 2) or after 14 days of dosing followed by 14 days of drug withdrawal (Expt 3).

### Behavioral Testing

For experiments, all mice were tested once in the same apparatus. Prior to testing, mice not on study were placed in the apparatus for some minutes before the experiment. The apparatus was cleaned with moist and dry tissues before testing each mouse. The aim was to create a low but constant background mouse odor for all experimental subjects. Testers were blind to the genotype and treatment during all testing and data analysis

### Open Field

The open-field apparatus was used to test multiple processes including anxiety/hyperactivity and habituation to a novel environment. The apparatus was a gray PVC-enclosed arena 50 × 9 × 30 cm divided into a 10 × 10 cm grid. Mice were brought to the experimental room 5–20 min before testing. A mouse was placed into a corner square facing the corner and observed for 3 min. The number of squares entered by the whole body (locomotor activity) and rears (both front paws off the ground, but not as part of grooming) were counted. The latency to the first rear was noted. The movement of the mouse around the field was recorded with a video tracking device for 3 min (version NT4.0, Viewpoint). The latency for the mouse to enter the brightest, central part of the field, total time spent in this central region, and total activity (as path length in cm) were recorded.

### Social Interaction

The apparatus was a test arena/cage roughly the same size as the home cage. Typically, this was a 40 × 23 × 12 cm cage, with a Perspex lid to facilitate viewing the mice. The arena had fresh wood chips on the floor. Non-experimental mice were introduced to the cage and then removed before starting the test to provide a background mouse odor. Mice were transferred to the experimental room 10–15 min prior to test to wake them up. Simultaneously, both a test subject and a juvenile were placed in the test cage. The total duration and number of bouts of social investigation, defined as sniffing and close following (<2 cm from the tail) of the stimulus juvenile, were measured for 3 min.

### Nesting

The test was performed in the same individual cages as above. Normal bedding covered the floor to a depth of 0.5 cm. Each cage was supplied with a “Nestlet,” a 5 cm square of pressed cotton batting (Ancare). Mice were placed individually into the nesting cages 1 hr before the dark phase, and the results were assessed the next morning. The nests were assessed on a 5-point scale, and the amount of un-torn Nestlet was weighed. Nest building was scored on a 5 point scale.

Score 1: The Nestlet was largely untouched (>90% intact).

Score 2: The Nestlet was partially torn up (50–90% remaining intact).

Score 3: The Nestlet was mostly shredded but often there was no identifiable nest site: <50% of the Nestlet remained intact but <90% was within a quarter of the cage floor area, i.e. the cotton was not gathered into a nest but spread around the cage. Note: the material may sometimes be in a broadly defined nest area but the critical definition was that 50–90% had been shredded.

Score 4: An identifiable, but flat nest: >90% of the Nestlet was torn up, the material was gathered into a nest within a quarter of the cage floor area, but the nest was flat, with walls higher than mouse body height (curled up on its side) on less than 50% of its circumference.

Score 5: A (near) perfect nest: >90% of the Nestlet was torn up, the nest was a crater, with walls higher than mouse body height on more than 50% of its circumference.

### Marble Burying

Transparent plastic cages were filled with a 10-cm deep layer of sawdust on top of which 10 glass marbles were placed in two rows. Each animal was left undisturbed in such a cage for 30 min, after which the number of marbles that were buried to at least two-thirds of their depth was recorded.

### Statistical Analysis of Behavioral Data

Data were analyzed by two-way analysis of variance (ANOVA) followed by post-test comparisons where appropriate using Tukey’s Multiple Comparison Test. Data are represented as the mean and standard error of the mean (SEM). Statistical analyses were performed in GraphPad Prism 7.03.

### Ballistic dye labeling and microscopy

Ballistic dye labeling (DiI and DiO) was performed according to protocols developed by Afraxis to label target neurons (Afraxis, San Diego, CA). Sections were slide mounted and cover slipped. Laser-scanning confocal microscopy (Olympus FV1000) was performed using a 63X objective (1.42 NA) to scan individually labeled neurons at high resolution (0.098 × 0.098 × 0.33 μm voxels). Target neurons were identified in the brain region of interest by anatomical location and cell morphology. Microscopy was performed blind to experimental conditions. 10 mice were tested in each experimental condition. A minimum of 5 cells were sampled from each mouse.

### Dendritic spine analysis, database lock and unblinding

Blind deconvolution (AutoQuant) was applied to raw three-dimensional digital images which were then analyzed for spine density and morphology by trained analysts. Individual spines were measured manually for (a) head diameter, (b) length, and (c) neck thickness from image Z-stacks using custom-built Afraxis ESP software. Each dendrite was analyzed by (on average) 2–3 independent analysts. Automated image assignment software distributed images to analysts in a randomized manner and ensured that each analyst performed measurements of near equal numbers of dendrites per group. Analysts were blinded to all experimental conditions (including treatment, brain region, and cell type). Statistical analysis of interanalyst variability for each dendrite was examined on-line and used to eliminate dendrites that did not meet interanalyst reliability criteria: For spine density and spine morphological classification, data across analysts were averaged to report data for each dendrite. Population distributions of each measure were compiled for each dendritic sample and pooled by group. Quantitative comparisons using the non-parametric Kolmolgorov-Smirnov test were used to assess group differences (Fig. 5). Considering the sensitivity of pooled-population non-parametric statistics, a conservative hypothesis test was applied (α = 0.0001). Population distributions for combinations of measures (spine length x head diameter or spine length x head diameter / neck width) were compiled for each dendritic sample and pooled by group. Difference maps were constructed by subtraction of raw distributions (control subtracted from target) as in Fig. 4. A 12-category classification scheme that describes highly granulated dendritic spine phenotypes was used to categorize every spine (Sup Fig. 8 & 9). These categories were collapsed into three categories representing immature, intermediate, and mature spine morphologies (Sup Fig. 8 & 9). Finally, an assessment independent from the 12-point scheme was used to describe classic spine phenotypes (e.g. mushroom, stubby, etc.).

All Afraxis experimenters were fully blinded to treatment conditions during the collection, assembly, and interpretation of the data. The database was locked after all spine data was collected prior to unblinding by treatment for statistical analysis.

### Statistical Analysis of Spine Morphometry

Values are reported in tables and plots as group means ± standard errors of the mean (SEMs) where each data value (individual mouse) represents the mean value of all segments sampled from a given mouse. For all group comparisons of parametric values, statistical significance was determined using the analysis of variance test (ANOVA; SPSS). Post-hoc comparisons were assessed using the Student’s t-test (2-tailed). For the spine analysis, non-parametric comparisons of individual measure population distributions were conducted using the 2-sample Kolmogorov-Smirnov test (α = 0.0001).

### Data Availability

The datasets generated during and/or analyzed during the current study are available from the corresponding author on reasonable request.

## Electronic supplementary material

Supplemental figures
