# Supplementary material for: Multiple Behavior Phenotypes of the Fragile-X Syndrome Mouse Model Respond to Chronic Inhibition of Phosphodiesterase-4D (PDE4D)
Source: Sci Rep. 2017 Nov 7;7:14653. doi: 10.1038/s41598-017-15028-x (PMC5677090; doi:10.1038/s41598-017-15028-x)

**Multiple Behavior Phenotypes of the Fragile-X Syndrome Mouse Model Respond to Chronic Inhibition of Phosphodiesterase-4D (PDE4D)**

Abbreviated title: Therapeutic Benefit of PDE4D Inhibition in Fragile-X Syndrome

Mark E. Gurney[1], Patricia Cogram[2], Robert M Deacon[3], Christopher Rex[4] and Michael Tranfaglia[5]

[1] Tetra Discovery Partners, Inc. Grand Rapids, MI, USA
[2] Fraunhofer Chile Research Foundation, Santiago, Chile
[3] Afraxis, Inc. San Diego, CA, USA
[4] FRAXA Research Foundation, Newburyport, MA, USA

## SUPPLEMENTAL FIGURES

**Supplemental Figure 1 |** Wild-type (WT) or *fmr1* KO (KO) adult male mice were gavaged with vehicle or BPN14770 for 14 days (Expt 1). Prior to preparation for spine morphometry, open field activity (Number of Squares crossed) was assessed 2 hr after the last dose. Data were analyzed by two-way ANOVA followed by Tukey's Multiple Comparison Test. There was a highly significant interaction between treatment and genotype ($F_{(1,36)}$ = **45.45**, *p<0.001*). P-values shown are: n.s. not significant, ** p <0.05, ** p<0.01, *** p<0.001* (*N* = 10 mice per group).

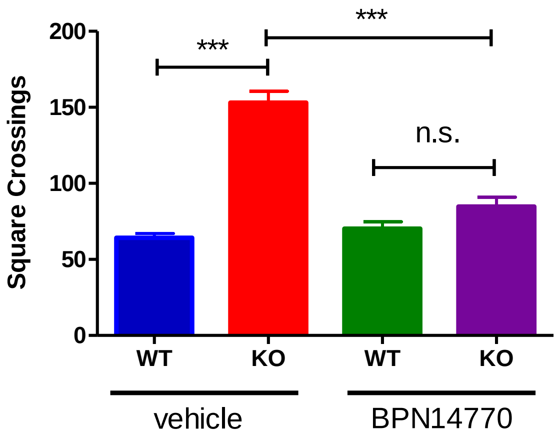

**Supplemental Figure 2 |** Spine morphometry in medial frontal cortex of fmr1 KO mice treated with vehicle (Vehicle) or BPN14770 (Treated) for 14 days as in Supplemental Figure 1. A 12-category classification scheme that describes highly granulated dendritic spine phenotypes was used to categorize every spine (below). These categories were collapsed into three categories representing immature (IMM), intermediate (INT), and mature (MAT) spine morphologies (middle panel). Finally, an assessment independent from the 12-point scheme was used to describe classic spine phenotypes; these categories were filopodia (F), long thin (LT), mushroom (M) and stubby (ST). Total spine density values are shown for each dendritic segment. Data were analyzed per animal. For all group comparisons of parametric values, statistical significance was determined using the analysis of variance test (ANOVA; SPSS). Post-hoc comparisons were assessed using the Student's t-test (2-tailed). P-values were ** <0.1; # <0.05.*

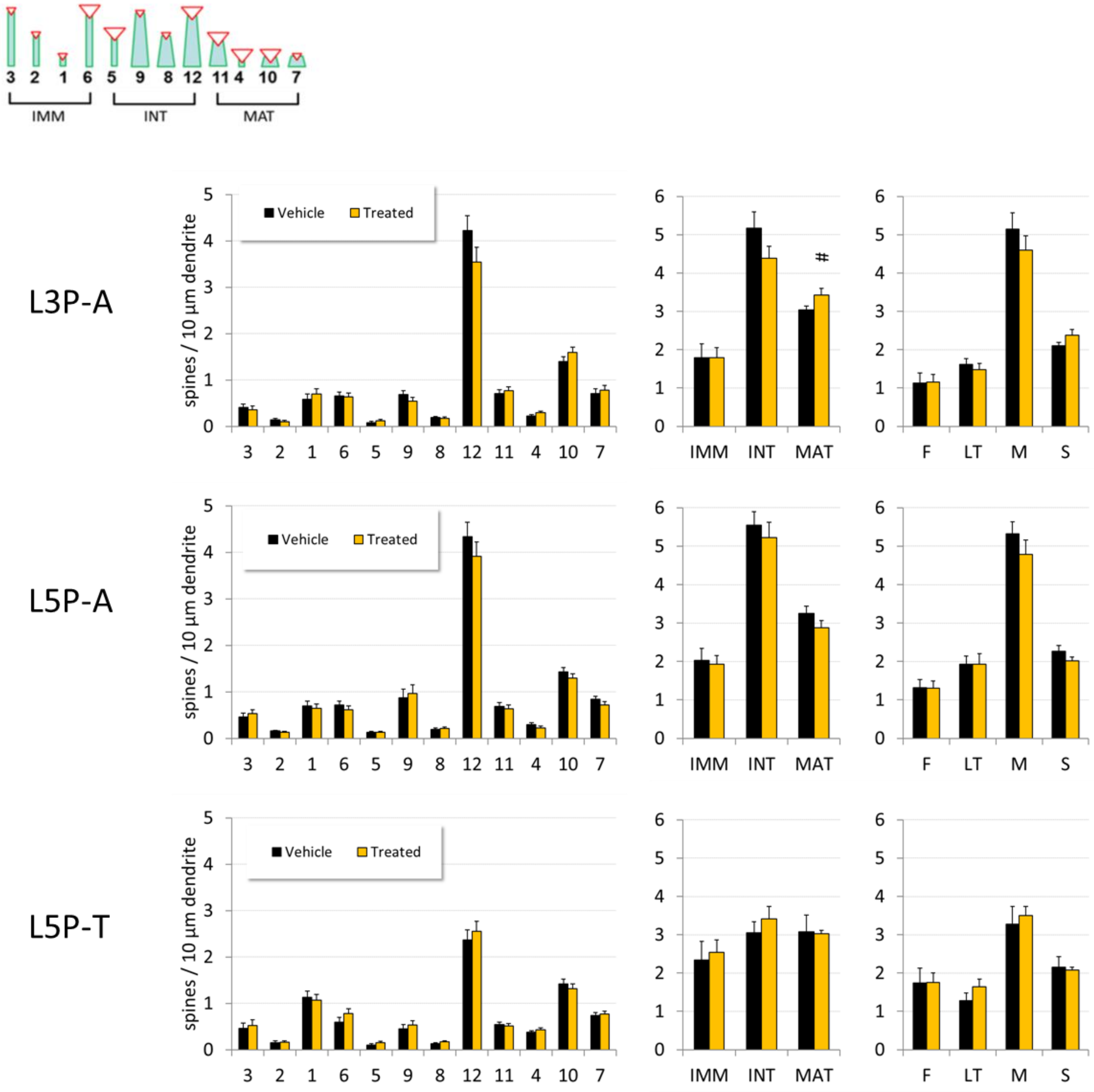

**Supplemental Figure 3 |** Spine morphometry in medial frontal cortex of fmr1 KO mice treated with vehicle (Vehicle) or BPN14770 (Treated) for 14 days as in Supplemental Figure 1. A 12-category classification scheme that describes highly granulated dendritic spine phenotypes was used to categorize every spine (below). These categories were collapsed into three categories representing immature (IMM), intermediate (INT), and mature (MAT) spine morphologies (middle panel). Finally, an assessment independent from the 12-point scheme was used to describe classic spine phenotypes; these categories were filopodia (F), long thin (LT), mushroom (M) and stubby (ST). Total spine density values are shown for each dendritic segment. Data were analyzed per dendrite. For all group comparisons of parametric values, statistical significance was determined using the analysis of variance test (ANOVA; SPSS). Post-hoc comparisons were assessed using the Student's t-test (2-tailed). P-values were ** <0.1; # <0.05.*

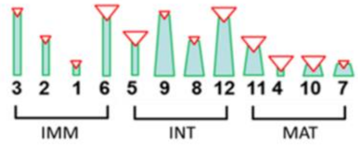


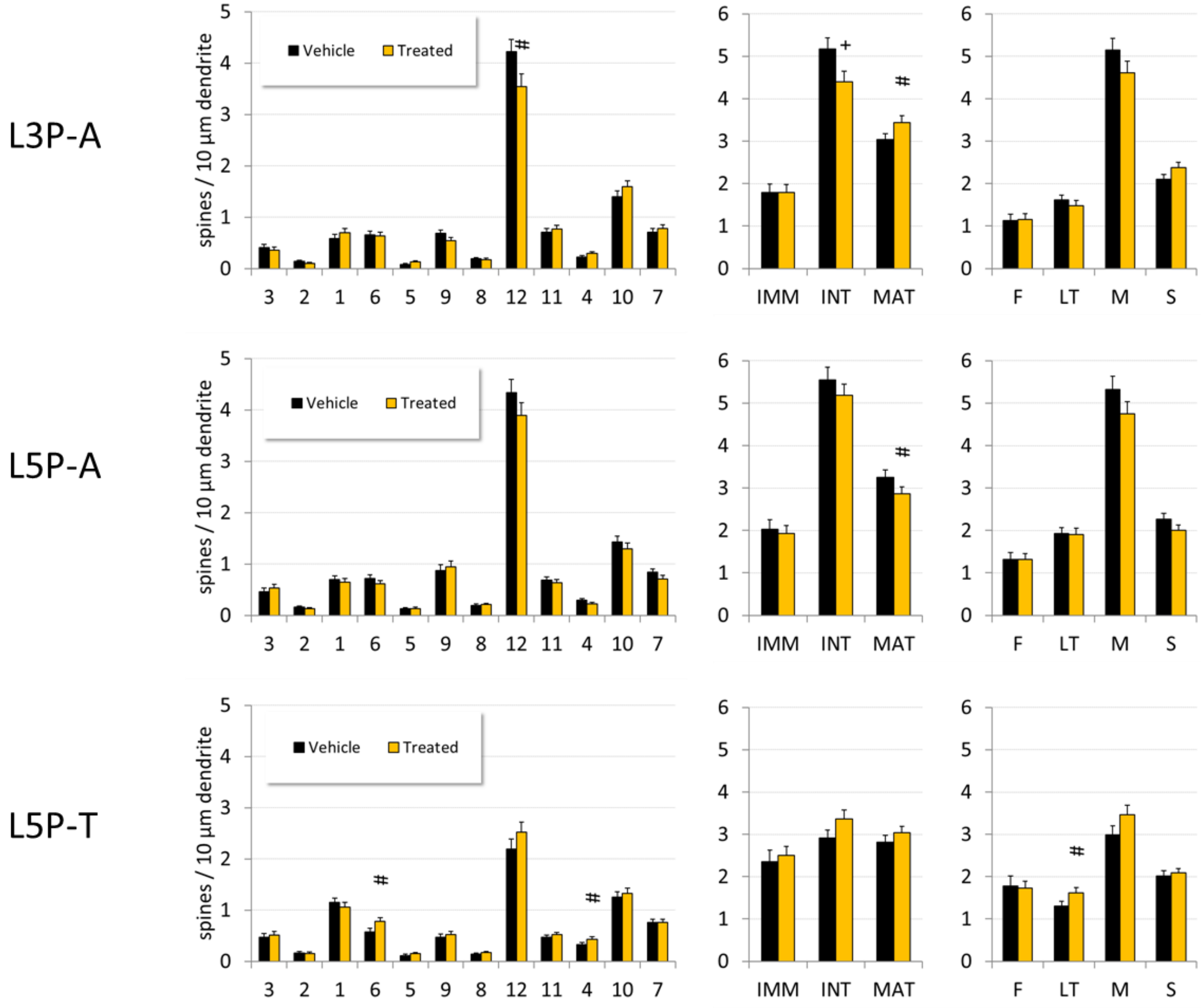

Supplement: Supplementary file 1 — Supplemental figures [file 41598_2017_15028_MOESM1_ESM.pdf]
